# Impact of marital status on overall survival in patients with early-stage hepatocellular carcinoma

**DOI:** 10.1038/s41598-022-14120-1

**Published:** 2022-11-19

**Authors:** Fangjie Chen, Ying Wu, Hong’en Xu, Tao Song, Senxiang Yan

**Affiliations:** 1 Zhejiang Chinese Medical University, Hangzhou, 310053 Zhejiang People’s Republic of China; 2 Department of Nursing, Zhejiang Provincial People’s Hospital, Affiliated People’s Hospital, Hangzhou Medical College, Hangzhou, 310014 Zhejiang People’s Republic of China; 3 Cancer Center, Department of Radiation Oncology, Zhejiang Provincial People’s Hospital, Affiliated People’s Hospital, Hangzhou Medical College, Hangzhou, 310014 Zhejiang People’s Republic of China; 4 Department of Radiation Oncology, The First Affiliated Hospital, Zhejiang University School of Medicine, Hangzhou, 310003 Zhejiang People’s Republic of China

**Keywords:** Cancer, Gastroenterology, Medical research, Oncology, Risk factors

## Abstract

The purpose of the present research was to assess the prognostic impact of marital status in hepatocellular carcinoma (HCC) patients with tumors ≤ 2 cm (stage Ia) based on the data from the Surveillance, Epidemiology, and End Results (SEER) database. Patients who received a histopathologic HCC diagnosis between 2004 and 2016 were recruited. Overall survival (OS) was the major outcome measure. The Cox regression model and the Fine-Gray regression model were used for the purpose of comparing and examining the prognostic value of marital status for OS. The data for a total of 2446 stage Ia HCC patients were extracted from the database. The median overall survival time was 96.0 months, with 5-year and 10-year overall survival rates of 58.2% and 45.8%, respectively. In both the Fine-Gray regression model and Cox regression model, marital status [married vs. unmarried and others, both *P* < 0.001, hazard ratio (HR) = 1.389 for Cox and HR = 1.378 for Fine-Gray], age at diagnosis, tumor grade, and surgery at the primary site independently served as prognostic indicators associated with OS. In conclusion, positive marital status was independently associated with better OS for stage Ia HCC patients, and its prognostic influence should be validated in the near future.

## Introduction

Primary liver cancer has been ranked as the seventh most prevalent malignant neoplasm and the second major contributor to cancer-related deaths on a global scale^1–3^. Among all types of liver cancers, hepatocellular carcinoma (HCC) is the dominant type and accounts for more than 75% of primary liver cancers^4,5^. Benefiting from early detection and timely treatment of some major risk factors, including alcoholic and/or nonalcoholic fatty liver disease and chronic HCV and/or HBV infection, the incidence rate of HCC has recently slowed in some areas^6–8^. Additionally, encouraging clinical results over the past three decades indicate that the overall survival (OS) rate of HCC has increased slightly^9,10^. Further, curative surgical resection, including liver transplantation or local ablation, for treating early-stage HCC has made great contributions^11^.

In addition, amid all of the indicators to defeat this disease, marital status has long been explored as a significant prognostic variable in a wide range of tumors. In 2013, Aizer et al. examined the influences of marital status on clinical outcomes among 10 major contributors to cancer-associated fatality in the United States using the Surveillance, Epidemiology and End Results (SEER) program^12^. For liver or intrahepatic bile duct cancers specifically, their results demonstrated that married patients exhibited a greater possibility of receiving definitive therapy (*P* < 0.01) and have significant survival benefits (*P* < 0.01) than unmarried persons (including divorced/separated, widowed, and single). However, in another large sample retrospective analysis conducted in Italy, a decreased risk of liver cancer was observed in unmarried patients^13^. Similarly, for HCC patients with poor or anaplastic differentiation who underwent surgical resection, a 2019 SEER report suggested that marital status had a non-significant benefit on survival outcomes^14^.

Considering that HCC patients with tumors ≤ 2 cm (stage Ia) could enjoy long-term survival and the unknown correlation between stage Ia HCC and marital status, we extracted and analyzed data from the SEER database to further evaluate the influences of marital status on survival status in this setting.

## Methods

### Patient selection

**Figure 1:**
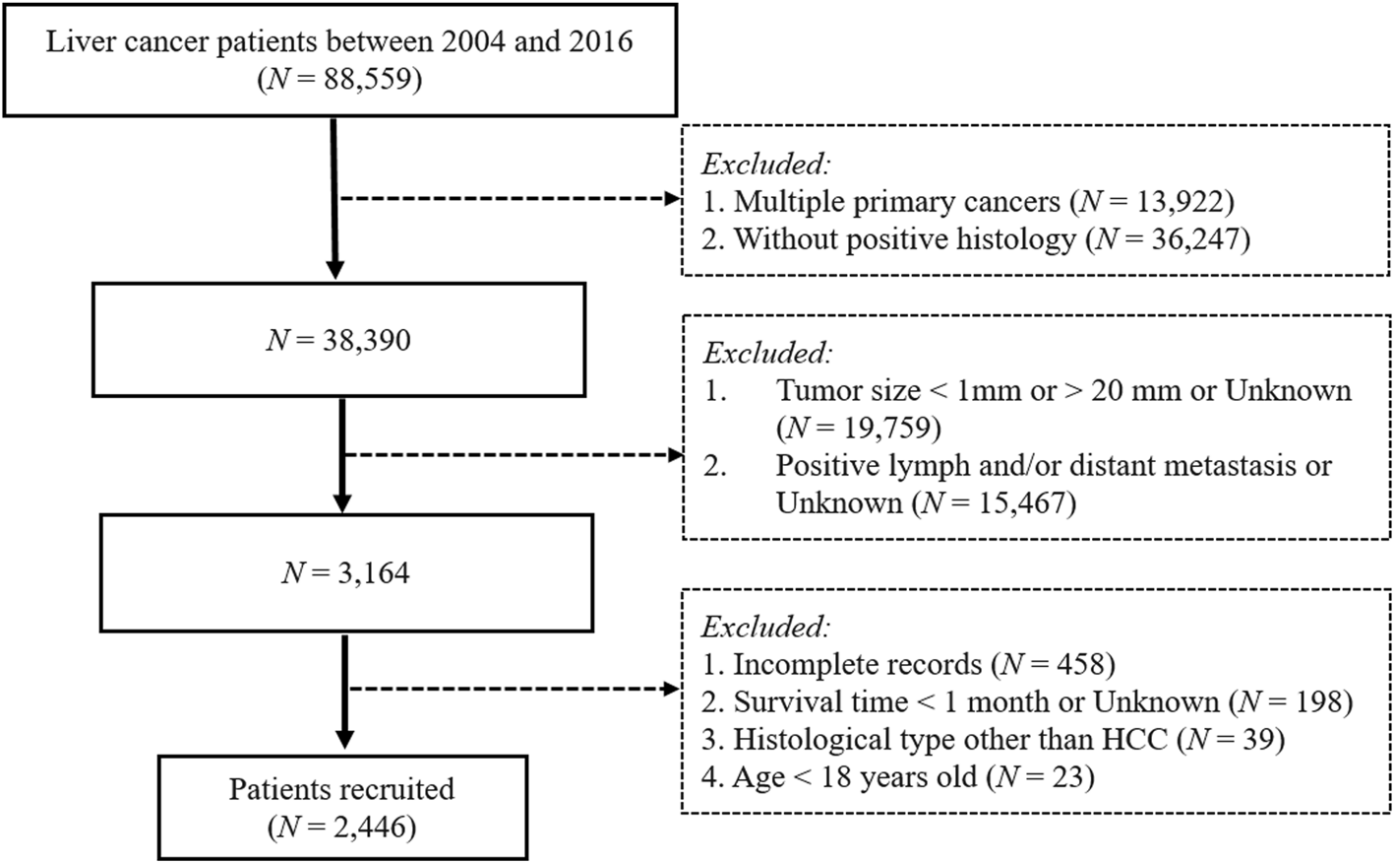
Patient selection flowchart.

This retrospective study obtained data from the SEER 18 population-based registries (SEER* Stat 8.3.6), which includes approximately 30% of the United States population. Data of 88,559 patients with confirmed liver cancer diagnoses from 2004 to 2016 was obtained from the database (ICD-O-3 Histology recodes 8140-8389). The following were the inclusion criteria used in recruiting participants for the present study: (1) patients who have a histopathological diagnosis of HCC; (2) tumors less than 2 cm without lymph node metastasis; and (3) upon HCC diagnosis, the patient must be no younger than 18 years of age. Below were some of the major exclusion criteria: (1) patients’ records of other cancers or with metastatic diseases and for whom HCC was not the first diagnosed cancer, and (2) HCC patients whose survival time was less than 1 month or who had untraced data (Fig. 1).

### Determination of study variables and outcomes

We queried the database for patient information including demographics, treatment history and outcomes (age, sex, race, marital status, local surgery, use of radiotherapy (RT), use of chemotherapy (CT), survival data including status (alive or dead), cause of death (COD) and COD to site recode, survival time in months, and cause-specific death classification). For HCC patients registered in 2016, eligible tumors were restaged according to the definitions in the AJCC 8th edition. We defined marital status as a binary factor by dividing married (including common law) by unmarried and others, which included divorced, separated, single (never married), widowed, unmarried or domestic partners, and unknown coded in the SEER data, as has been reported in other studies^15–17^. The items COD to site recode and cause-specific death classification indicated if a patient died from HCC (HCC-DSD: disease-specific death) or of causes other than HCC (HCC-NDSD: nondisease-specific death).

### Data analysis

As the main endpoint in the present research, we measured OS, which was defined as the duration between the day of HCC diagnosis and the day of death or the final follow-up recorded in the database. CSS (cause-specific survival) was described as the duration between the day of initial diagnosis and the point of death attributable to HCC or the date of the final follow-up recorded in the program, whichever came first. Patients’ baseline characteristics were summarized by descriptive statistics and frequency tables. One-way analysis of variance (ANOVA) was used to compare the proportions of different groups. The non-linear correlation between age at diagnosis and all CODs was investigated by means of a restricted cubic spline (RCS)^18,19^. Methods for multivariate and univariate analysis by Cox regression model were described in our previous studies^20,21^. In addition, considering that stage Ia HCC patients could enjoy long-term survival, we further regarded HCC-NDSD as a competing event in this cohort. In this model, the cumulative incidence function (CIF) was employed to determine the possibility of each factor in the univariate analysis and was checked with Gray’s test. According to the results of the univariate analysis, variables with a *p*-value < 0.05 were selected and incorporated into a multivariate competing-risks survival analysis with the aid of a proportional subdistribution hazard model, as determined by the Fine-Gray test. Hazard ratios (HRs) and their 95% confidence intervals (CIs) were also calculated in the analysis. A two-sided *P* value < 0.05 was established as a criterion of statically significant difference. Furthermore, to better illustrate the effects of marital status on liver cancer, the following librarians/databases were searched to trace eligible studies: Ovid MEDLINE®, PubMed, and Google Scholar updated until 31 December 2021 (present in the discussion section). The search strategy was developed and consisted of 2 main concepts: (1) liver cancer and (2) marital status. Methods for article selection were also described in our previous studies^22,23^ and we confirmed that the selection was performed in accordance with the PRISMA guidelines for systematic reviews.

The R software (version: 3.6.2; Institute for Statistics and Mathematics, Vienna, Austria; https://www.r-project.org) and SPSS software (version: 25.0 IBM Corporation, Armonk, NY, USA) was utilized to perform all analyses of statistical data.

### Ethics statement

It was not necessary to get written informed consent for participating in the present research as the information contained in the SEER database has been de-identified and is publically available following authorization. The present research was exempted from ethical assessment by the Institutional Review Board of Zhejiang Provincial People’s Hospital. We hereby certify that the present research was conducted in conformity with the Declaration of Helsinki.

## Results

### Demographic and baseline characteristics

**Figure 2:**
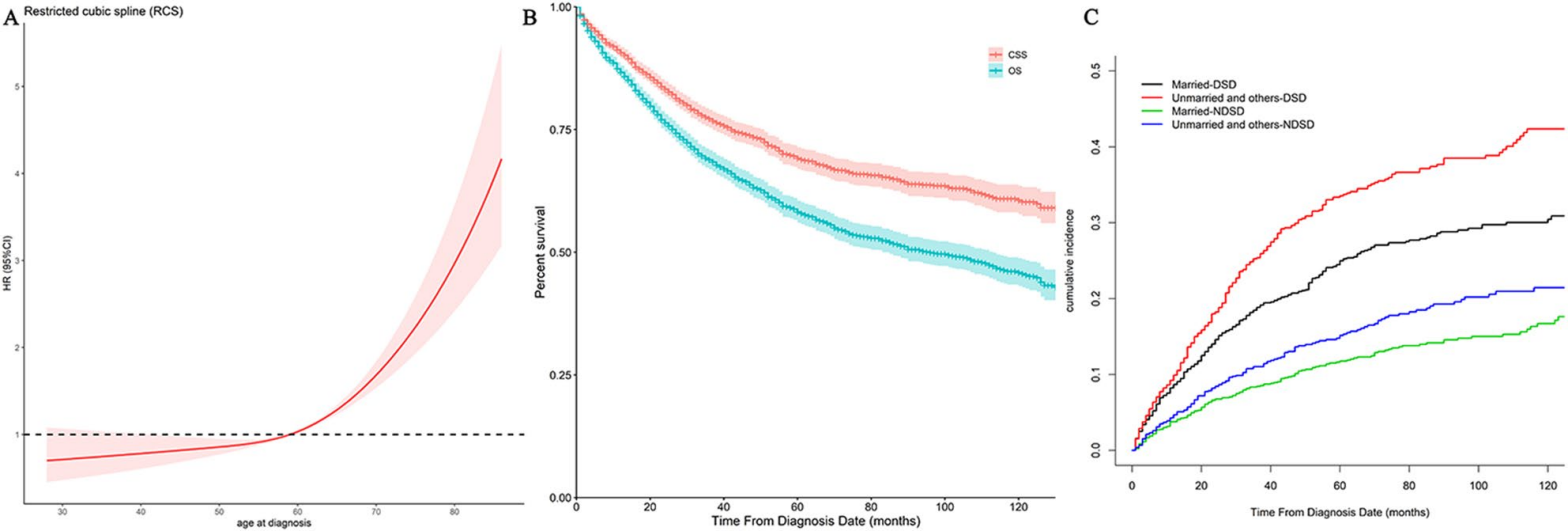
(**A**) A restricted cubic spline (RCS) demonstrated that 59 years old was a reasonable cutoff point. (**B**) OS and CSS of stage Ia HCC patients. (**C**) The cumulative risk curves according to marital status.

We extracted the data from 2446 eligible stage Ia HCC patients from the SEER database between 2004 and 2016. Table 1 presents the demographics and baseline features of the patients who were included in the present research. The ages of patients at diagnosis ranged between 20 and 90 years old with 59 years old as the median age. We further applied RCS with 3 knots (5th, 50th, and 95th centiles) to evaluate the association between age at diagnosis and all CODs (Fig. 2A). Based on the result, the appropriate inflection point to age at diagnosis was also 59 years old. Among all enrolled patients, 1397 (57.1%) patients were married, and 1049 (42.9%) HCC patients were classified as unmarried or other. A total of 1860 (76.0%) of the patients underwent cancer surgery. Only 64 (2.6%) HCC patients were treated with RT. The correlation coefficient of different variables is presented in Fig. S1. No factors were highly correlated with other baseline characteristics. Additionally, male patients were observed to have significant higher proportion with positive marital status than unmarried and others (45.0% vs. 29.4%, *P* < 0.001), while age had no significance with marital status.

**Table 1:** Baseline characteristics of stage Ia HCC patients.

Characteristic	ALL (%)	HCC-DSD (%)	HCC-NDSD (%)
**Age (years)**			
< 59	1182 (48.3)	271 (41.5)	156 (47.9)
≥ 59	1264 (51.7)	382 (58.5)	170 (52.1)
**Race**			
White	1763 (72.1)	476 (72.9)	240 (73.6)
Non-white	683 (27.9)	177 (27.1)	86 (26.4)
**Sex**			
Female	626 (25.6)	186 (28.5)	72 (22.1)
Male	1820 (74.4)	467 (71.5)	254 (77.9)
**Marital status**			
Married	1397 (57.1)	327 (50.1)	165 (50.6)
Unmarried and others	1049 (42.9)	326 (49.9)	161 (49.4)
**Differentiation**			
Well and moderately differentiated	1556 (63.6)	336 (51.5)	213 (65.3)
Poorly and undifferentiated	164 (6.7)	61 (9.3)	20 (6.1)
Unknown	726 (29.7)	256 (39.2)	93 (28.6)
**Surgery at the primary site**			
No/unknown	586 (24.0)	282 (43.2)	84 (25.8)
Cancer-directed surgery performed	1860 (76.0)	371 (56.8)	242 (74.2)
**Radiotherapy (RT)**			
No/unknown	2382 (97.4)	638 (97.7)	319 (97.9)
Yes	64 (2.6)	15 (2.3)	7 (2.1)
**Chemotherapy (CT)**			
No/unknown	1792 (73.3)	444 (68.0)	259 (79.4)
Yes	654 (26.7)	209 (32.0)	67 (20.6)

HCC: Hepatocellular carcinoma; DSD: disease-specific death; NDSD: non disease-specific death.

### Survival outcomes

The OS rates for 5- and 10-year were 58.2% (95% CI, 0.560–0.604) and 45.8% (95% CI, 0.431–0.485), respectively, with a median OS time of 96.0 months (95% CI, 82.920–109.080, Fig. 2B). Of the 2,446 HCC patients, 326 patients died of HCC-NDSD, such as other infectious and parasitic diseases including HIV (n = 122), other causes of death (n = 39), and diseases of the heart (n = 38), accounting for 61.0% of the total (Fig. S2). Table 1 presents the baseline features of HCC patients who died due to HCC-DSD and HCC-NDSD. The CSS rates over 5 and 10 years were 69.1% (95% CI, 0.669–0.713) and 60.6% (95% CI, 0.577–0.635), correspondingly, with a median CSS time not achieved at the time of analysis (Fig. 2B). Among married patients, the median OS duration was 130.0 ± 7.7 months (95% CI, 114.961–145.039), and in patients classified as unmarried and other, the median OS duration was 65.0 ± 5.6 months (95% CI, 53.951–76.049).

### Fine-gray regression analysis

A univariate analysis using Fine-Gray test suggested that age at diagnosis (*P* < 0.001), sex (*P* = 0.004), marital status (*P* < 0.001), surgery at the primary site (*P* < 0.001), tumor differentiation (*P* < 0.001), and CT (*P* < 0.001) significantly impacted the prognosis of stage Ia HCC patients. The cumulative risk curves for marital status are shown in Fig. 2C. The CIF was found to be elevated over 36, 60, and 120 months and was elevated for advanced age, female sex, unmarried and others, poorly or undifferentiated or unknown tumor grade, no or unknown status of surgery at the primary site, and receiving CT among eight variables. The CIF values among married HCC patients were 18.8%, 25.0%, and 30.6% at 36, 60, and 120 months, respectively. The corresponding figures for patients recoded as unmarried and others were 25.4%, 33.5%, and 42.4%, correspondingly. Table 2 depicts the findings recorded from the CIF values and univariate analysis.

**Table 2:** Univariate analysis in stage Ia HCC patients by using a competing risk model.

			Cumulative incidence function (CIF)		
Factors	Gray's test	*P*-value	36-months	60-months	120-months
**Age (years)**	35.156	< 0.001			
< 59			0.178	0.239	0.286
≥ 59			0.253	0.334	0.442
**Race**	0.618	0.432			
White			0.231	0.290	0.347
Non-white			0.179	0.276	0.376
**Sex**	8.128	0.004			
Female			0.252	0.328	0.412
Male			0.205	0.273	0.337
**Marital status**	20.163	< 0.001			
Married			0.188	0.250	0.306
Unmarried and others			0.254	0.335	0.424
**Differentiation**	57.738	< 0.001			
Well and moderately differentiated			0.167	0.227	0.295
Poorly and undifferentiated			0.322	0.395	0.427
Unknown			0.297	0.386	0.466
**Surgery at the primary site**	250.974	< 0.001			
No/unknown			0.456	0.538	0.614
Cancer-directed surgery performed			0.142	0.209	0.278
**Radiotherapy (RT)**	0.398	0.528			
No/unknown			0.214	0.285	0.355
Yes			0.323	0.323	0.323
**Chemotherapy (CT)**	13.425	< 0.001			
No/unknown			0.195	0.260	0.332
Yes			0.273	0.358	0.413

We then employed the six variables that had statistical significance in the univariate analysis entered into the Fine-Gray model. According to the findings from the Fine-Gray regression model, age at the time of diagnosis (< 59 vs. ≥ 59, *P* < 0.001, HR = 1.419, 95% CI: 1.246–1.618), marital status (married vs. unmarried and others, *P* < 0.001, HR = 1.378, 95% CI: 1.212–1.568), tumor differentiation (well or moderately differentiated vs. poorly or undifferentiated, *P* = 0.007, HR = 1.402, 95% CI: 1.095–1.795; well or moderately differentiated vs. unknown, *P* = 0.032, HR = 1.169, 95% CI: 1.014–1.349), surgical resection of the primary site (no/unknown vs. yes, *P* < 0.001, HR = 0.340, 95% CI: 0.293–0.393) and treatment of CT (no/unknown vs. yes, *P* = 0.009, HR = 0.819, 95% CI: 0.706–0.952) all served as prognostic indicators that were significantly associated with OS in an independent manner (Table 3). Furthermore, multivariate analysis of NDSD also indicated that marital status (married vs. unmarried and others, *P* < 0.001, HR = 1.481, 95% CI: 1.308–1.680) was an independent factor associated with NDSD (Table S1).

**Table 3:** Multivariate analysis of OS in stage Ia HCC patients with the Cox regression model and the Fine-Gray regression model.

	Cox regression analysis				Fine‐Gray regression analysis			
Factor	*P* value	HR	95% CI	95% CI	*P* value	HR	95% CI	95% CI
			Lower	Upper			Lower	Upper
Age, < 59 versus ≥ 59	< 0.001	1.415	1.244	1.611	< 0.001	1.419	1.246	1.618
Race, white versus non-white	–				–			
Sex, female versus male	0.581	0.960	0.830	1.110	0.564	0.958	0.829	1.108
Marital status, married versus unmarried and others	< 0.001	1.389	1.223	1.578	< 0.001	1.378	1.212	1.568
Grade, reference: well and moderately								
Poorly and undifferentiated	0.005	1.398	1.106	1.767	0.007	1.402	1.095	1.795
Unknown	0.055	1.146	0.997	1.318	0.032	1.169	1.014	1.349
Surgery at the prim site, no/unknown versus yes	< 0.001	0.357	0.311	0.409	< 0.001	0.340	0.293	0.393
RT, no/unknown versus yes	–				–			
CT, no/unknown versus yes	–				0.009	0.819	0.706	0.952

HR, Hazard ratio; CI, confidence interval.

### Cox regression analysis

According to the results of the Cox regression analysis, four clinicopathological characteristics, namely age at the time of diagnosis, sex, tumor differentiation, marital status, and one treatment-related parameter (surgery at the primary site) were significantly associated with OS (Table S2). The findings recorded from the multivariate analysis illustrated that the significant covariates were age at the time of diagnosis (< 59 vs. ≥ 59, *P* < 0.001, HR = 1.415, 95% CI: 1.244–1.611), marital status (married vs. unmarried and others, *P* < 0.001, HR = 1.389, 95% CI: 1.223–1.578), tumor differentiation (well or moderately differentiated vs. poorly or undifferentiated, *P* = 0.005, HR = 1.398, 95% CI: 1.106–1.767; well or moderately differentiated vs. unknown, *P* = 0.055 HR = 1.146, 95% CI: 0.997–1.318) and surgical resection of the primary site (no/unknown vs. yes, *P* < 0.001, HR = 0.357, 95% CI: 0.311–0.409; Table 3). Both the Fine-Gray regression model and the Cox regression model demonstrated that marital status independently served as a prognostic indicator for OS.

## Discussion

The objective of the present research was to evaluate the effect of marital status on OS in stage Ia HCC patients since the influence of marital status remains controversial in this setting. While using available data in the SEER database, we demonstrated that positive marital status acted as a prognostic variable in an independent manner favoring improved OS in both the Fine-Gray regression model and Cox regression model.

The results exploring the effects of marital status on liver cancer patients are summarized in Table 4 following chronological order^14,24–36^. Fourteen studies that met the eligibility requirements were enrolled. It should be noted that marital status was not covered in the National Cancer Data Base, which is also a widely used database for the analysis of various malignancies^37^. Most studies (13/14, 92.9%) were retrospective analyses with data extracted from the SEER database, with only one report having external validation with patients from their own cancer center. Among the 13 reports, 3 studies showed no significant association between marital status and survival outcomes, one study investigated the benefit of RT in HCC patients with major vascular invasion (*P* = 0.834 in univariate analysis), one study assessed the impact of RT in unresectable HCC patients (*P* = 0.475), and the other study evaluated the effects of marital status on patients developing less differentiated HCC who underwent surgical resection (*P* = 0.370). The remaining 10 studies all supported the benefit of positive marital status for liver cancer patients despite different situations of patient enrollment. A similar study was reported by Peters and colleagues^29^. In this study, 13,694 HCC patients diagnosed with stage I-II disease were enrolled in the present research. Positive marital status was first demonstrated to have a significantly higher likelihood of patients receiving liver resection, and liver transplantation (both *P* < 0.001). The findings recorded from the Cox regression analysis correlated with DSS, being married was shown to have a significantly longer DSS (*P* = 0.010; HR = 0.71; 95% CI, 0.55–0.92). In the only prospective study^36^, Chiu et al. compared four models predicting quality of life (QoL) following hepatic resection in 332 stage I-III HCC patients during 2012–2015 at three institutions. The findings indicated that marital status was one of nine independent variables involved in the model for predicting the QoL scores obtained six months after hepatic resection.

**Table 4:** Summary of published studies on the impact of marital status on liver cancer.

Reference (First author)	No. of patients and study design	Study period (year)	Cancer type (HCC/ICC/Both)	Source of data (IE/Database)	Main study population and age cutoff value (CV)	Main conclusion on marital status
Chen et al.^24^	1,352, retrospective	1973–2013	ICC	SEER	Patients stratified by localized/Regional/Distant stage, CV = 70	Positive for CSS*
Zhang et al.^25^	8,621, retrospective	1988–2007	HCC	SEER	Patients with all stages, receiving hepatic resection, CV = 45, 60, 75	Positive for CSS
Wu et al.^26^	13,408, retrospective	1998–2013	HCC	SEER	Patients with all stages, receiving hepatic resection, CV = 60	Positive for CSS
He et al.^27^	40,809, retrospective	2004–2012	Both	SEER	Patients stratified by localized/Regional/Distant stage, CV = 60	Positive for OS and CSS
Wu et al.^28^	12,168, retrospective	2004–2012	HCC	SEER	Patients with all stages stratified by race and gender, CV = 60, 80	Positive for OS and CSS
Peters et al.^29^	13,694, retrospective	2004–2012	HCC	SEER	Stage I-II HCC, CV = 60	Positive for DSS
Lin et al.^30^	3,181, retrospective	2004–2013	HCC	SEER	Patients with major vascular invasion receiving radiotherapy, CV = 65	Negative for OS
Zhang et al.^31^	1,305, retrospective	2004–2014	HCC	SEER	Unresected HCC patients with all stages receiving radiotherapy, CV = 50,60,70,80	Negative for OS
Xiao et al.^32^	15,638, retrospective	2004–2014	HCC	SEER and IE	Patients with all stages cxcluded unknown baseline characterics, CV = 60, 70, 80	Positive for OS and CSS
Yan et al.^14^	1,581, retrospective	2004–2015	HCC	SEER	All stages with less differentiated HCC receiving hepatic resection, CV = 60	Negative for CSS
Wu et al.^33^	8,677, retrospective	2010–2012	Both	SEER	Compared according to matastatic status, CV = 60, 75	Positive for OS and CSS
Guo et al.^34^	1,567, retrospective	2010–2014	HCC	SEER	Patients with bone metastatis, CV, not available	Positive for OS
Liang et al.^35^	4,933, retrospective	2010–2015	Both	SEER	Patients with all stages without hepatic resection, CV = 60, 75	Positive for CSS
Chiu et al.^36^	332, prospective	2012–2015	HCC	IE (three centers)	Stage I-III HCC receiving hepatic resection, median age = 60	Positive for QoL

HCC: Hepatocellular carcinoma; ICC: intrahepatic cholangiocarcinoma; IE: institutional experience; *: statistically significant for regional disease; QoL: quality of life.

Unfortunately, all of these enrolled studies concluded that marital status on survival outcomes was based on the findings revealed by the Cox proportional hazard models. In fact, for HCC patients diagnosed with stage Ia, 33.3% (326/979) of patients died due to various reasons other than HCC, as revealed in the current study (Fig. S2). This nonhomogeneity could certainly cause bias in calculating the effects of marital status on OS. Starting with this consideration and the knowledge that stage Ia HCC patients could enjoy prolonged survival duration, with a 10-year overall survival rate of 45.8%, we then employed the Fine-Gray regression model according to the findings recorded from the CIF analysis. Marital status still acted as prognostic parameter affecting OS in an independent manner (*P* < 0.001, HR = 1.378). Previously, Yang et al. compared the Fine-Gray regression model with the Cox regression model in penile cancers^38^. Survival analysis indicated that the findings derived with the aid of the Cox regression model were different from those obtained by the Fine-Gray regression model, while the Kaplan–Meier curve analysis led to an overestimation compared to the CIF analysis of penile cancer patients. Similar findings were also observed in esophageal cancer^39^ and cecum cancer^40^ in the literature. Therefore, when comparing survival outcomes among different variables, especially for competing events in survival outcomes, it is worth considering the possibility of CIF analysis to avoid overestimation results^41^.

This study is first limited by its inherent retrospective nature, with some heterogeneity in the analysis. Some important information, such as patients’ baseline characteristics, including lesbian, gay, bisexual, transgender, and queer status, liver function, alcoholic and/or nonalcoholic fatty liver disease, HBV ± HCV infection, cancer location, Child–Pugh score, and treatment complications, was not available in the SEER database. Secondly, the SEER database only recorded marital status at patients’ initial diagnosis, and changes in marital status might occur during the long-term follow-up periods, which could alter the influence of marital status. Third, a high incidence of receiving CT was observed within the cohort, which was mainly explained by these patients were re-staged based on the newly AJCC 8th staging manual. In previous guidelines for treating early stage HCC with/without microvascular invasion, adjuvant CT followed by surgical resection was recommended. Finally, potential interactions among sex, generation and socioeconomic status might influence marital status, and whether the findings generated in the current study apply to other populations or demographics around the world, need to be confirmed by large, well-designed, prospective studies in the future.

In conclusion, we evaluated the impact of marital status on the OS outcomes of HCC patients with tumors ≤ 2 cm registered in the SEER database between 2004 and 2016. Through comparison between two different regression models, positive marital status can be used to act as a prognostic indicator for better OS outcomes in an independent manner. Our results are consistent with earlier major studies supporting the benefit of being married for stage Ia HCC patients. When treating localized diseases with potential medical cures, additional interventions, including but not limited to family and social support, should be given to subpopulations with negative marital status.

## Supplementary Information

Supplementary Information 1.

Supplementary Information 2.

Supplementary Information 3.

Supplementary Information 4.

Supplementary Information 5.

## Data Availability

The datasets produced for this work (SEER database) are accessible via the following link: https://seer.cancer.gov/data/access.html. Further inquiries can be directed to the corresponding authors.
