# Supplementary material for: Impact of marital status on overall survival in patients with early-stage hepatocellular carcinoma
Source: Sci Rep. 2022 Nov 19;12:19923. doi: 10.1038/s41598-022-14120-1 (PMC9675859; doi:10.1038/s41598-022-14120-1)
Supplement: Supplementary file 1 — Supplementary Information 1. [file 41598_2022_14120_MOESM1_ESM.docx]

**Supplementary figure legends**

Figure S1. The correlation coefficients among different variables.

Figure S2. Causes of death (COD) other than HCC registered in the database.
