# Supplementary figures and images for: Impact of marital status on overall survival in patients with early-stage hepatocellular carcinoma
Source: Sci Rep. 2022 Nov 19;12:19923. doi: 10.1038/s41598-022-14120-1 (PMC9675859; doi:10.1038/s41598-022-14120-1)

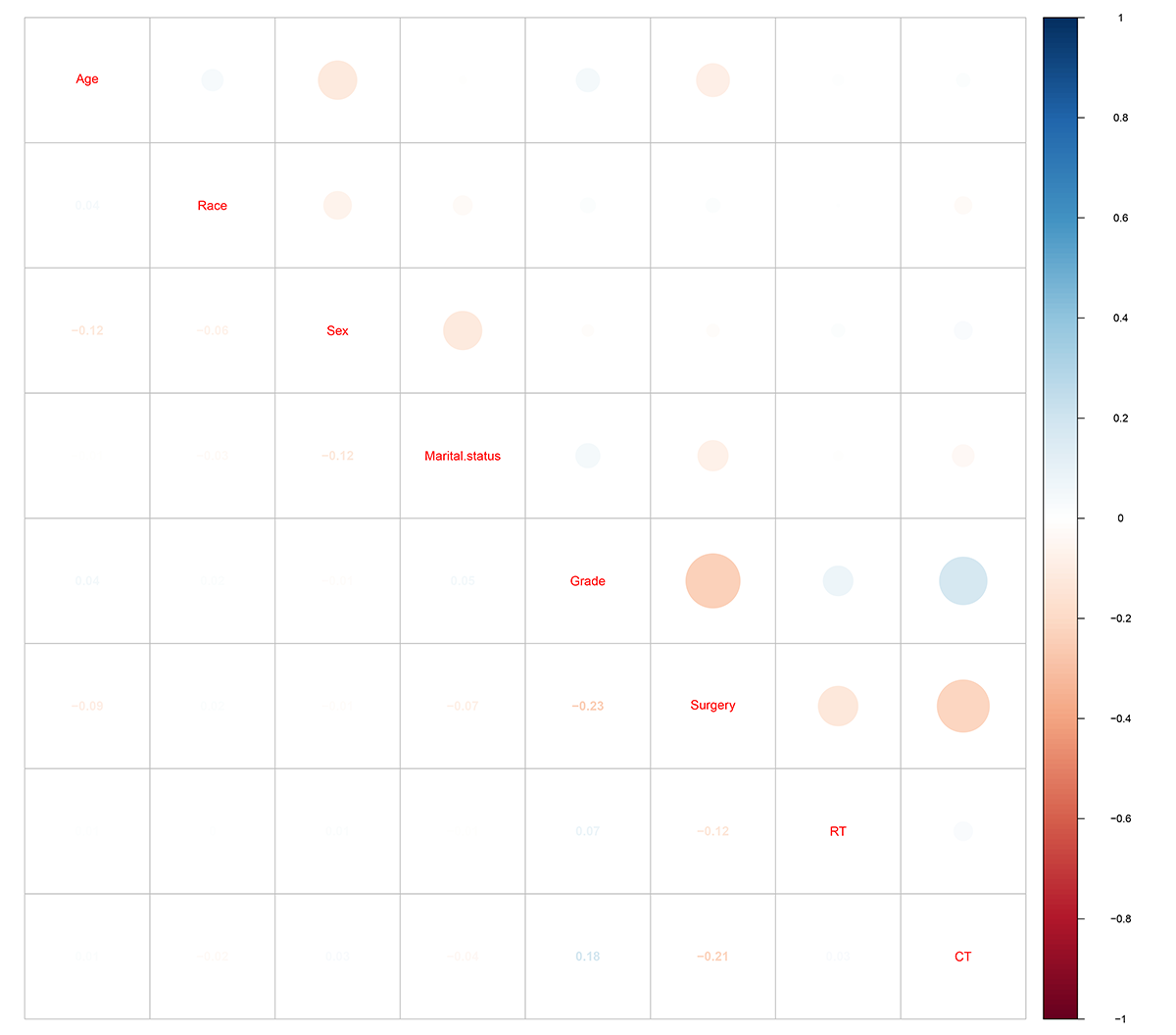

Supplement: Supplementary file 2 — Supplementary Information 2. [file 41598_2022_14120_MOESM2_ESM.tif]

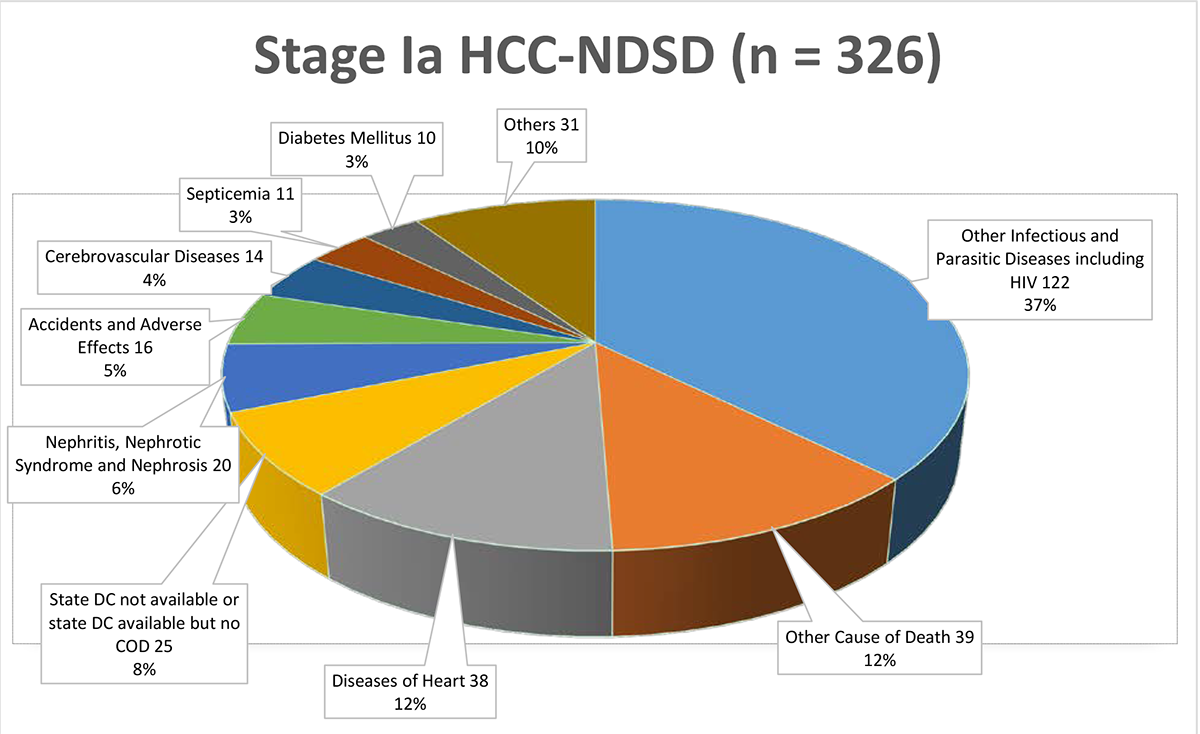

Supplement: Supplementary file 3 — Supplementary Information 3. [file 41598_2022_14120_MOESM3_ESM.tif]
