# Supplementary material for: Impact of marital status on overall survival in patients with early-stage hepatocellular carcinoma
Source: Sci Rep. 2022 Nov 19;12:19923. doi: 10.1038/s41598-022-14120-1 (PMC9675859; doi:10.1038/s41598-022-14120-1)
Supplement: Supplementary file 4 — Supplementary Information 4. [file 41598_2022_14120_MOESM4_ESM.docx]

Table S1. Univariate and multivariate analysis of NDSD in stage Ia HCC patients by using the Fine-Gray regression model.

|  | Univariate analysis | | | | |  | Multivariate analysis | | | |
| --- | --- | --- | --- | --- | --- | --- | --- | --- | --- | --- |
|  |  |  | Cumulative incidence function (CIF) | | |  | *P*-value | HR | 95% CI Lower | 95% CI Upper |
| Factors | Gray's test | *P*-value | 36-months | 60-months | 120-months |  |  |  |  |  |
| *Age (years)* | 2.566 | 0.109 |  |  |  |  | - |  |  |  |
| < 59 |  |  | 0.090 | 0.123 | 0.175 |  |  |  |  |  |
| ≥ 59 |  |  | 0.100 | 0.142 | 0.201 |  |  |  |  |  |
| *Race* | 0.417 | 0.519 |  |  |  |  | - |  |  |  |
| White |  |  | 0.098 | 0.133 | 0.190 |  |  |  |  |  |
| Nonwhite |  |  | 0.090 | 0.129 | 0.180 |  |  |  |  |  |
| *Sex* | 0.953 | 0.329 |  |  |  |  | - |  |  |  |
| Female |  |  | 0.085 | 0.107 | 0.167 |  |  |  |  |  |
| Male |  |  | 0.099 | 0.140 | 0.193 |  |  |  |  |  |
| *Marital status* | 7.394 | 0.007 |  |  |  |  |  |  |  |  |
| Married |  |  | 0.084 | 0.118 | 0.167 |  |  | 1.000 |  |  |
| Unmarried and others |  |  | 0.110 | 0.151 | 0.214 |  | < 0.001 | 1.481 | 1.308 | 1.680 |
| *Differentiation* | 1.294 | 0.524 |  |  |  |  | - |  |  |  |
| Well and moderately differentiated |  |  | 0.094 | 0.136 | 0.198 |  |  |  |  |  |
| Poorly and undifferentiated |  |  | 0.098 | 0.114 | 0.133 |  |  |  |  |  |
| Unknown |  |  | 0.098 | 0.128 | 0.178 |  |  |  |  |  |
| *Surgery at the primary site* | 2.559 | 0.110 |  |  |  |  | - |  |  |  |
| No/unknown |  |  | 0.128 | 0.159 | 0.192 |  |  |  |  |  |
| Cancer-directed surgery performed |  |  | 0.086 | 0.124 | 0.185 |  |  |  |  |  |
| *Radiotherapy (RT)* | 0.102 | 0.750 |  |  |  |  | - |  |  |  |
| No/unknown |  |  | 0.100 | 0.176 | 0.176 |  |  |  |  |  |
| Yes |  |  | 0.095 | 0.131 | 0.187 |  |  |  |  |  |
| *Chemotherapy (CT)* | 6.936 | 0.008 |  |  |  |  |  |  |  |  |
| No/unknown |  |  | 0.082 | 0.112 | 0.133 |  |  | 1.000 |  |  |
| Yes |  |  | 0.100 | 0.139 | 0.205 |  | 0.066 | 0.879 | 0.766 | 1.008 |
