# Supplementary material for: Impact of marital status on overall survival in patients with early-stage hepatocellular carcinoma
Source: Sci Rep. 2022 Nov 19;12:19923. doi: 10.1038/s41598-022-14120-1 (PMC9675859; doi:10.1038/s41598-022-14120-1)
Supplement: Supplementary file 5 — Supplementary Information 5. [file 41598_2022_14120_MOESM5_ESM.docx]

Table S2. Univariate analysis of OS in stage Ia HCC patients by using the Cox regression model.

|  | Univariate analysis | | | |
| --- | --- | --- | --- | --- |
| *Factor* | ***P*** *value* | *HR* | *95% CI Lower* | *95% CI Upper* |
| *Age, < 59 vs. ≥ 59* | < 0.001 | 1.530 | 1.347 | 1.738 |
| *Race, White vs. Non-white* | 0.195 | 0.911 | 0.791 | 1.049 |
| *Sex, Female vs. Male* | 0.050 | 0.867 | 0.752 | 1.000 |
| *Marital status, Married vs. Unmarried and others* | < 0.001 | 1.473 | 1.300 | 1.670 |
| *Differentiation, reference: Well and moderately differentiated* | < 0.001 |  |  |  |
| Poorly and undifferentiated | 0.001 | 1.497 | 1.185 | 1.890 |
| Unknown | < 0.001 | 1.513 | 1.323 | 1.731 |
| *Surgery at the primary site, No/unknown vs. Yes* | < 0.001 | 0.328 | 0.288 | 0.374 |
| *Radiotherapy (RT), No/unknown vs. Yes* | 0.409 | 0.837 | 0.548 | 1.277 |
| *Chemotherapy (CT), No/unknown vs. Yes* | 0.126 | 0.897 | 0.780 | 1.031 |

HR, hazard ratio; CI, confidence interval.
